# Evaluating the prevalence of psychiatric comorbidities associated with pediatric scoliosis utilizing ResearchMatch

**DOI:** 10.1007/s43390-024-00926-8

**Published:** 2024-08-12

**Authors:** Jeffrey W. Chen, Stefan W. Koester, Campbell Liles, Stephen Gannon, Christopher M. Bonfield

**Affiliations:** 1 Department of Neurological Surgery, Baylor College of Medicine, Houston, TX USA; 2 School of Medicine, Vanderbilt University, Nashville, TN USA; 3 Surgical Outcomes Center for Kids, Monroe Carell Jr. Children’s Hospital, Nashville, TN USA; 4 Department of Neurological Surgery, Vanderbilt University Medical Center, Nashville, TN USA

**Keywords:** Scoliosis, Spinal deformity, National survey, Psychiatry, Depression, Anxiety

## Abstract

**Purpose:**

The goal of this study is to characterize the self-reported prevalence of psychiatric comorbidities among patients with adolescent scoliosis.

**Methods:**

Eligible patients across the US were surveyed using ResearchMatch, a validated online platform. The survey collected patient demographics, type of scoliosis, scoliosis treatment received, and the mental health diagnoses and interventions.

**Results:**

Nearly all (98%) of the 162 respondents were patients themselves, the remainder of which were parents. The majority of whom were female (93%), Caucasian (85%), and diagnosed with idiopathic scoliosis (63%). The median age of diagnosis was 13 (IQR 11–18). Most respondents had mild to moderate scoliosis (65%), and 17% received surgical treatment. 76 of 158 (48%) responded that scoliosis affected their overall mental health, and 92 (58%) had received a mental health diagnosis-76% were diagnosed after their scoliosis diagnosis. Of the 92 with mental health diagnoses, the most common diagnoses were clinical depression (83%), anxiety (71%), negative body image (62%). Over 80% of patients received medical treatment or therapy. Of those with depression, 38.4% received counseling and 45.2% received medication. 52% of the respondents also had immediate family members with mental health diagnoses, with siblings (48%) having the highest proportion.

**Conclusion:**

According to the CDC, the prevalence of US teenagers with diagnosed depression was found to be 3.9% and anxiety disorder to be 4.7%, notably higher among adolescent girls. In this national sample, over half of adolescent scoliosis patients report psychiatric comorbidity, often diagnosed years later. The most prevalent psychiatric condition is depression, anxiety, and body-image disturbances. These findings highlight the importance of awareness of the psychiatric impact of adolescent scoliosis, and importance of screening and treatment of comorbid mental health conditions.

**Level of evidence:**

IV.

## Introduction

Pediatric scoliosis is relatively common among children, with 2–3% meeting criteria for diagnosis. There are multiple types of scoliosis, including neuromuscular scoliosis, cerebral palsy, muscular dystrophies, neurofibromatosis, and congenital scoliosis, the most common being adolescent idiopathic scoliosis (AIS) [1]. While scoliosis may be equally distributed among males and females, females are at higher risk for larger curves and needing treatment [2–4].

Spine deformities can severely affect self-perception, relationships, and mental health, which have been well documented in the adolescent and adult population [5, 6]. Various professional societies including the Scoliosis Research Society, American Academy of Orthopedic Surgeons, and Society on Scoliosis Orthopedic and Rehabilitation Treatment recommend AIS screening in early adolescent years [7]. Aside from the esthetic changes to the growing adolescent body brought on by scoliosis, treatment itself can also be a source of psychologic distress [8–10]. Non-operative treatment like bracing may require young children to spend 12 to 20 h of their day in a brace, with longer bracing times being more effective [11, 12]. Such bracing can lead to restriction in physical activities or cause self-isolation in fear of being rejected by peers. On the other hand, spinal deformity corrective surgery in adolescents with AIS can have a mixed effect on anxiety and depression [13, 14]. Most patients have improved body image immediately post-operatively, but anxiety and depression about concerns regarding visual difference have been shown to emerge later.

The link between psychologic comorbidities and pediatric scoliosis have been noted in some studies [5, 6] however, there have been few surveys that assess a large population on a national level. Large, multicenter studies are limited in the specific questions that can be asked [15], while single-center studies lack generalizability [16]. Therefore, the goal of this study is to survey patients and characterize the prevalence of self-reported psychologic impact of scoliosis diagnosis among pediatric patients using ResearchMatch.

## Methods

### Survey design and distribution

A survey was designed to extensively document the prevalence of psychiatric comorbidities among pediatric scoliosis patients. Questions asked basic demographics, including sex, race, ethnicity, state of residence, family size, income, and insurance. Scoliosis history was also noted, asking questions regarding the age of diagnosis, curvature severity, and non-surgical or surgical management. Finally, the survey asked questions about the diagnosis of psychiatric conditions (depression, anxiety, body-image dysphoria, and eating disorders), its onset, and related management. The survey was designed, administered, and recorded through REDCap as de-identified data [17].

This online-survey study was distributed to eligible patients and parents of patients with pediatric-diagnosed scoliosis using ResearchMatch, a validated online-survey platform connecting academic institutions across the United States. Patients receive the survey based on their diagnosis, and voluntarily respond to the questions. ResearchMatch has been vetted by the Clinical and Translational Science Awards Informatics Key Function Committee and approved for funding by the National Institutes of Health’s National Center for Research Resources [18]. ResearchMatch, a disease-neutral, online recruitment registry that facilitates connecting match individuals with the specific disease who wish to participate in clinical research studies with researchers actively searching for volunteers across the United States [18, 19] Patients with a diagnosis of pediatric scoliosis and enrolled in ResearchMatch registry were eligible and received an electronic survey, which was completed voluntarily. The research design and survey questionnaire were approved by IRB#211023 and design was approved by the study ethics council.

### Statistical analysis

Data aggregation and exploratory analysis were performed using R, version 4.0.1 (R Foundation for Statistical Computing, Vienna, Austria). Means, standard deviations, interquartile ranges (IQR), and percentages were calculated for characteristics and patient demographics. Subgroup analyses were performed for patients with idiopathic scoliosis vs non-idiopathic causes, as well as patients diagnosed between 8 and 12 vs those diagnosed later. A power analysis for two independent means was calculated to find the number of patients necessary in each group to detect 95% power, and based on an expected mean difference 5 points, the power analysis determined that at least 44 patients would be required per group.

## Results

A total of 174 respondents were included in this study, 170 (98%) being the patient themselves. Most respondents were females (*n* = 161, 92.5%) and Caucasian (*n* = 151, 86.7%). Responses were received from respondents across all 50 states. The median household income was between $50,000–$74,999, and over half (*n* = 100, 59%) of the respondents had private or commercial insurance. Table 1 provides a table of the demographics of the study cohort.

**Table 1:** Demographics of respondent from ResearchMatch

Characteristic	No. responses	Result
Evaluated for scoliosis	162	161 (99%)
Clinically diagnosed with scoliosis	162	162 (100%)
Who is filling out this survey?	162	
I have/had scoliosis		158 (98%)
My child has/had scoliosis		4 (2.5%)
Participant’s biologic sex	158	
Female		147 (93.0%)
Male		11 (7.0%)
Child’s biological sex	4	
Female		2 (50%)
Male		2 (50%)
Participant’s race/ethnicity	162	
Native American/Alaska Native		6 (3.7%)
East Asian/Asian American		4 (2.5%)
South Asian/Indian American		2 (1.2%)
Native Hawaiian/Other Pacific Islander		1 (0.6%)
Black/African American/Afro-Caribbean		8 (4.9%)
Middle Eastern/North African/Arab American		3 (1.9%)
White/Caucasian		138 (85%)
Hispanic/Latino/Spanish origin		6 (3.7%)
Other		2 (1.2%)
Prefer not to answer		2 (1.2%)
Child’s race/ethnicity	4	
Native American/Alaska Native		0 (0%)
East Asian/Asian American		0 (0%)
South Asian/Indian American		0 (0%)
Native Hawaiian/Other Pacific Islander		0 (0%)
Black/African American/Afro-Caribbean		1 (25%)
Middle Eastern/North African/Arab American		0 (0%)
White/Caucasian		3 (75%)
Hispanic/Latino/Spanish origin		0 (0%)
Other		0 (0%)
Prefer not to answer		0 (0%)
Reported number of siblings the participant has	158	
0		19 (12%)
1		51 (32%)
2		48 (30%)
3 +		40 (25%)
Reported number of siblings the child has	4	
0		1 (25%)
1		1 (25%)
2		2 (50%)
Approximate yearly household income?	160	
$0–$24,999		29 (18%)
$25,000–$49,999		41 (26%)
$50,000–$74,999		27 (17%)
$75,000–$99,999		20 (12%)
$100,000–$149,999		27 (17%)
$150,000–$199,999		8 (5.0%)
$200,000 +		8 (5.0%)
Type of scoliosis were you/your child diagnosed with	158	
Idiopathic scoliosis (e.g., adolescent idiopathic scoliosis)		100 (63%)
Neuromuscular scoliosis		17 (11%)
Congenital scoliosis		20 (13%)
Other		21 (13%)
Primary state of residence	162	
Westcoast		38 (23.5%)
South		58 (35.8%)
Northeast		27 (16.7%)
Midwest		38 (23.5%)
Other territory		1 (0.6%)

Statistics presented: *n* (%); mean (SD)

Westcoast—CA, NV, OR, WA, ID, MT, WY, CO, UT, AZ

South—NM, TX, OK. AR, LA. MS, AL, GA, FL, NC, SC, VA, WV, KY, TN

Midwest—ND, SD, NE, KS, MN, IA, IA, MO, IL, IN, WI, MI, OH

Northeast—ME, VT, NH, MA, CT, RI, NJ, NY, PA, DE, MD

The main type of scoliosis reported was idiopathic (*n* = 108, 64%), followed by congenital (*n* = 21, 21%) and neuromuscular scoliosis (*n* = 17, 10%), with a mean age of diagnosis at 13 (IQR = 11–18) (Table 2). Most respondents replied that their scoliosis was on the spectrum of mild to moderate (64.2%). There were 64 respondents (38%) who underwent conservative treatment, and 33 respondents (19%) underwent surgical treatment, mostly fusions (93.3%). One patient had undergone a Chiari decompression. All patients who underwent surgical treatment underwent bracing as well. The scoliosis types and subsequent treatment are provided in Table 2.

**Table 2:** Scoliosis type and subsequent treatments

Characteristic	No. responses	Result
Age at which participant or participant’s child was diagnosed with scoliosis	156	18 (14); 13 (7)
Participant or participant’s child underwent a non-surgical treatment for scoliosis (e.g., brace)	158	59 (37.3%)
Severity of scoliosis	135	
Mild		39 (29%)
Mild to moderate		13 (9.6%)
Moderate		36 (27%)
Moderate to severe		11 (8.1%)
Severe		24 (18%)
Very severe		12 (8.9%)
Type of non-surgical treatment for scoliosis	69	
Brace		41 (59.4%)
Chiropractor		10 (14.5%)
Physical therapy/exercise		14 (20.3%)
Injections		1 (1.4%)
Shoe lift		2 (2.9%)
Electrical stimulation		1 (1.4%)
Age at which participant or participant’s child for the first non-surgical scoliosis-related treatment	58	14 (7); 12 (5)
Participant or participant’s child underwent a surgical operation to treat scoliosis	161	28 (17%)
Type of surgical treatment for scoliosis	23	
Instrumentation		23 (14.3%)
Chiari decompression surgery		1 (1.0%)
Laminectomy		1 (1.0%)
Age at which participant or participant’s child for the first scoliosis-related operation	28	19 (11); 15 (5)

Statistics presented: mean (SD); median (IQR) OR *n* (%)

Nearly half of respondents stated their overall mental health was affected by scoliosis (*n* = 86, 49.7%) (Table 3). There were 103 respondents who were diagnosed with a mental health disorder, 79 respondents (76.7%) of whom were diagnosed after their scoliosis diagnosis. The most common diagnosis was depression (*n* = 86, 83.4%), with the mean age of diagnosis at 24 ± 10 years. All respondents reported receiving treatment in the form of counseling (44.5%), behavioral therapy (12.9%), and/or medication (50%). A subgroup analysis of mental health when considering idiopathic scoliosis vs non-idiopathic scoliosis revealed a significantly lower age of depression diagnosis for the idiopathic group (22 ± 9 vs 27 ± 10, *p* = 0.02). An additional subgroup analysis of respondents with scoliosis diagnosed between ages 8 and 12 years revealed no significant difference in mental health metrics.

**Table 3:** Prevalence and onset of scoliosis on mental health

Characteristic	No. responses	Result
Overall mental health has been affected due to scoliosis	158	76 (48%)
Child’s overall mental health has been affected due to scoliosis	4	4 (100%)
Previously diagnosed with a mental health disorder	158	92 (58%)
Child previously diagnosed with a mental health disorder	4	2 (50%)
Diagnosed with a mental health disorder before or after being diagnosed with scoliosis	92	
Before		22 (24%)
After		70 (76%)
Child diagnosed with a mental health disorder before or after being diagnosed with scoliosis	2	
Before		0 (0%)
After		2 (100%)
Received a clinical diagnosis of depression	92	76 (83%)
Child received a clinical diagnosis of depression	2	1 (50%)
Age at which participant was first diagnosed with depression	76	25 (10); 21 (14.25)
Age at which child was first diagnosed with depression	0	0 (NA)
Participant received treatment/therapy for depression	76	75 (99%)
Child received treatment/therapy for depression	1	1 (100%)
Type of therapy/treatment received for depression	146	
Counseling		56 (38.4%)
Behavioral therapy		18 (12.3%)
Medication		66 (45.2%)
Hospitalization		4 (2.7%)
EMDR		1 (1.0%)
Alpha stimulation		1 (1.0%)
Type of therapy/treatment child received for depression		
Behavioral therapy	1	1 (100%)
Received a clinical diagnosis of an adjustment disorder (e.g., PTSD, behavioral issues)	91	31 (34%)
Child received a clinical diagnosis of an adjustment disorder (e.g., PTSD, behavioral issues)	2	1 (50%)
Age when participant was first diagnosed with adjustment disorder	31	94 (354); 30 (20.75)
Age when child was first diagnosed with adjustment disorder	1	3 (0)
Participant received treatment/therapy for adjustment disorder	31	29 (94%)
Child received treatment/therapy for adjustment disorder	1	1 (100%)
Type of therapy/treatment participant received for adjustment disorder	52	
Counseling		21 (40.4%)
Behavioral therapy		12 (23.1%)
Medication		13 (25.0%)
EMDR		5 (9.6%)
Service dog		1 (1.9%)
Type of therapy/treatment child received for adjustment disorder	1	
Behavioral therapy		1 (100%)
Participant received a clinical diagnosis of anxiety	92	65 (71%)
Child received a clinical diagnosis of anxiety	2	0 (0%)
Age at which participant first diagnosed with anxiety	65	57 (245); 21.5 (15.5)
Age at which child first diagnosed with anxiety	NA	NA
Participant received treatment/therapy for anxiety	65	64 (98%)
Child received treatment/therapy for anxiety	0	0 (NA%)
Type of therapy/treatment participant received for anxiety	112	
Counseling		39 (34.8%)
Medication		56 (50.0%)
Behavioral/psycho therapy		15 (13.4%)
Service dog		1 (0.9%)
EMDR		1 (0.9%)
Type of therapy/treatment child received for anxiety	0	0 (NA%)
Participant received a clinical diagnosis of an eating disorder (e.g., anorexia nervosa, bulimia nervosa)	92	12 (13%)
Child received a clinical diagnosis of an eating disorder (e.g., anorexia nervosa, bulimia nervosa)	2	0 (0%)
Age at which participant was first diagnosed with an eating disorder	64	26.2 (12.4); 21.5 (5–60)
Age at which child was first diagnosed with an eating disorder	0	0 (NA%)
Participant received treatment/therapy for an eating disorder	12	10 (83%)
Child received treatment/therapy for an eating disorder	0	0 (NA%)
Type of therapy/treatment participant received for an eating disorder	10	
Counseling		7 (70%)
Dietary adjustment		2 (20%)
Behavioral therapy		1 (10%)
Participant struggled with a negative body image (e.g., self-consciousness with appearance, poor self-image, altered body image)	92	57 (62%)
Child struggled with a negative body image (e.g., self-consciousness with appearance, poor self-image, altered body image)	2	0 (0%)
Age at which participant first diagnosed with a body image disorder	36	20 (11); 16 (12.5)
Age at which child first diagnosed with a body image disorder	0	0 (NA%)
Participant received treatment/therapy for a body image disorder	56	11 (20%)
Child received treatment/therapy for a body image disorder	0	0 (NA%)
Type of therapy/treatment received by participant for a body image disorder	12	
Counseling		10 (83.3%)
Medication		1 (8.3%)
Behavioral therapy		1 (8.3%)
Participant received a clinical diagnosis of any other mental health disorders	89	24 (27%)
Child received a clinical diagnosis of any other mental health disorders	2	1 (50%)
Other mental health disorders	33	
Obsessive compulsive disorder		8 (24.2%)
Attention deficit/hyperactivity disorder		6 (18.2%)
Bipolar disorder		6 (18.2%)
Personality disorder		2 (6.1%)
Alcoholism		1 (3.0%)
Post-traumatic stress disorder		1 (3.0%)
Social phobia		1 (3.0%)
Pervasive development disorder		1 (3.0%)
Autism		1 (3.0%)
Tourette’s		1 (3.0%)
Panic attack		1 (3.0%)
Trichotillomania		1 (3.0%)
Essential tremor		1 (3.0%)
Immediate family has been diagnosed with a mental health disorder	160	84 (52%)
Immediate family members who have received a mental health-disorder diagnosis	58	
Mother		22 (38%)
Father		7 (12%)
Sibling		28 (48%)
Maternal grandparent		1 (1.7%)
Child’s immediate family members who have received a mental health-disorder diagnosis	1	
Father		1 (100%)

Statistics presented: mean (SD); median(IQR) OR *n* (%)

The second most common mental health diagnosis was anxiety (*n* = 72, 71%). Like depression, anxiety was diagnosed at 26 ± 11 years and with nearly all (99%) seeking treatment in the form of counseling (34.1%), behavioral therapy (14.3%), and/or medication (50%).

The third most common mental health diagnosis was body-image disturbance (*n* = 63, 62%), which unlike depression and anxiety was diagnosed at a younger mean age of 19 ± 11 years. Far fewer respondents sought treatment (*n* = 11, 18%), and those who did mostly sought out treatment in the form of counseling (83.3%). Other common mental health diagnoses include adjustment-related disorders (34%), eating disorders (12%), obsessive–compulsive disorder (8%), attention-deficit/hyperactivity disorder (8%).

Of 149 respondents, 45 (30.2%) reported wearing a brace. Of those with use of a brace, a greater proportion reported being affected by mental health (31 (69%) vs 46 (44%), *p* = 0.006) as well as body-image disturbance (20 (77%) vs 30 (50%), *p* = 0.02). The age at which depression was diagnosed in the brace cohort was also lower in comparison to the non-brace cohort (20 ± 7 vs 25 ± 10, *p* = 0.03). The severity of scoliosis did not correlate with the reporting of a body-image disturbance. Finally, 93 (54%) of respondents also noted immediate family members with mental health disorders. Most commonly siblings (32/66, 48%) followed by mothers (26/66, 39%).

## Discussion

In this paper, we use an online platform to survey patients or parents of patients with pediatric-diagnosed scoliosis about the presence of other psychiatric diagnoses. Among 174 respondents, over half of patients (58%) reported a mental health-disorder diagnosis, most of whom received their diagnosis after their scoliosis diagnosis. Our survey further reveals that the most reported diagnosis is depression, followed by anxiety and negative body image. Compared to the general population, the rates of psychiatric comorbidities among pediatric scoliosis patients is much greater than that of the general youth population [15, 20–22]. A CDC survey study of US teenagers estimates the national prevalence of diagnosed depression to be 3.9% (95% CI 3.6–4.3%), and anxiety disorder to be 4.7% (4.4–5.1%) [20]. They found that depression was notably higher among older adolescent females, who had an 18.2% (95% CI 17.5–18.9%) for at least one lifetime major depressive episode. The prevalence among young adults (18–25) is comparable to adolescents, a trend that remains relatively unchanged after adjusting for sociodemographic characteristics [23].

In our study, the prevalence of any psychiatric comorbidity among pediatric scoliosis is higher relative to the average adolescent in the United States [23, 24]. In a longitudinal community study assessing the prevalence of adolescent pediatric psychiatric disorder in the general population, Costello et al. found that the cumulative risk of having at least one psychiatric disorder increases with age, with a higher prevalence of multiple diagnoses compounding upon one another, especially among girls [20]. Our study of pediatric scoliosis had a cohort primarily female, which may add to the risk factor of developing depression and anxiety. In our study, we also found that all patients had multiple psychiatric conditions simultaneously. While it is still unclear if these develop simultaneously or in sequence, some authors note that families of psychiatric comorbidities develop together in a phenomenon called “heterotypic continuity.” Costello et al. found heterotypic continuity to be pronounced in girls along the depression–anxiety spectrum, a finding also noted in our study.

### Mood disorders: depression and anxiety

In our study, we found 86% of those who had a mental health diagnosis had clinical depression that required treatment, of which 50% required treatment with medication. The prevalence of depression in this cohort is many folds higher than reported in the literature [24]. This finding reiterates the high prevalence of depression among juveniles and adolescents with scoliosis, which has been noted by other authors to be particularly pronounced among adolescent female patients [6, 25–27]. While this connection between pediatric scoliosis and mood disorders is well known, the spectrum of clinical presentation remains difficult to gage given the multifactorial nature of mood disorders. Plaszewski et al., in a cross-sectional study comparing 68 AIS patients with 76 non-AIS controls found the former group had a higher score on the beck depression index (45% vs 33%) [28].

Most of the self-reported scoliosis severity in this study was within the mild to moderate category. It remains debated whether larger physical deformities are associated with greater emotional distress. Contrary to expectation, some studies have found that the severity of scoliosis does not demonstrate a strong relationship with emotional and psychologic distress [29, 30]. On the other hand, authors like Pratt et al. [31], Glowacki et al. [32] and Misterska et al. [33] all found that larger thoracic spine humps worsen the perception of body image and heightened risk of emotional distress.

While some studies suggest that AIS patients have a higher prevalence of mood disorders, a robust standard measurement tool similar to that of the tool for body image or eating disorders is not available for these patients. In addition, it can be challenging to differentiate true mood disorders and confounders from the overall quality of life variations. For example, in Karakaya et al. patients with higher SR-22 scores, which indicate higher quality of life, demonstrated a positive association with self-esteem and a negative association with depression [34].

### Body image and eating disorder

Our study also found a high proportion of patients with body-image disturbances (62%), and to a lesser degree eating disorders (12%). Pediatric scoliosis is a particularly challenging entity that arises during a critical period of an adolescent’s physical and social development. Body-image disturbances have been reported to range from 35 to 50% of adolescent patients [9, 10], and are perhaps the most investigated psychiatric comorbidity associated with AIS.

Controversy exists regarding body-image disturbances following non-operative vs operative treatment. Koroveissis et al. reported that adolescents treated with braces report greater insecurity about their body image and are likely to worry about the lasting effect of scoliosis on their bodies [35]. Perhaps most significant about this study is that girls were 24 times more likely to feel ashamed about their bodies and 16 times more likely to think their bodies to be unattractive.

Despite the ongoing debate across the literature, there is no clear consensus on whether bracing or surgical management is superior in reducing body-image disturbances. From the work of Zhang et al., it seems that patients who receive surgical correction may have higher self-esteem than adolescents without surgical correction [36]. On the other hand, Miterska et al. found that pediatric scoliosis patients treated surgically felt a moderate level of stress related to body deformation, which was significantly higher than the stress reported by patients treated with bracing [37]. Bunge et al. find that having surgery after bracing compared to surgery alone led to lower self-image domains [38]. A review of 58 articles by Gallant et al., found that lower BMI and less curve correction have less favorable outcomes, and perhaps body image is key for patient outcomes, regardless of treatment modality [39].

A less commonly explored topic is eating disorders and pediatric scoliosis. Only 13% of respondents noted they had an eating disorder. Multiple authors have noticed that adolescents with idiopathic scoliosis have lower BMI when compared to matched controls [40–44]. Only Smith et al. and Zaina et al. explicitly discuss the incidence of eating disorders but conclude with different findings. The former found higher incidence of restrictive eating while the latter with a lower incidence [41, 43]. Cantele et al. in their study find that adolescent scoliosis and the use of brace treatment do not seem to be related with higher risk of developing eating symptomatology [45]. The most interesting finding they present is that higher cognitive function and level of independent function, increases the risk of psychiatric comorbidities. While distinct from eating disorders, body-image disturbances share a large overlap in characteristics and demographics [46, 47]. Given that pediatric scoliosis patients typically already demonstrate lower BMI, body fat, bone density, and lean muscle, eating disorders can compound an existing dangerous condition [41, 48]. Yet, despite what seems to be an obvious connection, few studies have clearly demonstrated increased eating disorders. New endocrine studies have emerged that implicate dysregulation of leptin and ghrelin signaling pathways among AIS patients [49–52].

### Treatment options

Treatment options can also greatly affect the development of psychiatric disorders. Treatment options and their effect on psychiatric disorders have been an area of interest. The goal of either bracing or surgical correction is to prevent further curvature, correct existing curvature, and improve self-image and restore health-related quality of life [53, 54]. Results of arthrodesis have demonstrated improved body image and pain levels [29, 55, 56]. Bracing, on the other hand, has been shown to have mixed results [57–60], with perhaps the most substantial evidence by Weinstein et al. in a randomized control study between bracing and observation demonstrating no difference in body image or quality of life [61]. In a 12-month prospective study for scoliosis patients treated with bracing, Glowacki et al. found that anxiety levels were highest at the first of three assessment [32]. A longer duration of bracing risked increased anxiety levels as well. LaMontagne et al., in their study, found that anxiety and pain were considered to be major concerns for scoliosis patients treated operatively [25]. On the other hand, Hawes indicated that AIS-related anxiety has been shown to result from insecurity about spinal deformity progression [26]. Many studies have examined the relationship between treatment options, both conservative and surgical on the effect of adolescent patients. Tao et al. [62] find that non-operative treatment like bracing can be a risk factor for depression. Specifically, they find that females, older age of treatment, negative parental attitudes, larger Cobb angles, among other factors can contribute to greater risks of depression.

### Family

Finally, the paper found that family members of pediatric patients with scoliosis also had a higher prevalence of mental health diagnosis. While this paper did not survey this further, the impact of a child’s diagnosis on family may affect family dynamics and even diminish the caregiver’s ability to function. This finding is consistent with the literature for not only scoliosis, but other congenital disorders that cause physical deformities. For example, caregivers of children with craniosynostosis, stress can negatively affect the psychosocial development of children [63, 64]. For scoliosis, Wang et al. in a cross-sectional survey in Chinese parents, found that caregivers were at risk of developing depression and anxiety, which was closely associated with children developing depression and anxiety as well [65]. In a review, Motyer et al. found that parents face challenges of acquiring knowledge and coping with a child undergoing invasive spinal surgery, both of which can contribute to worsening psychologic well-being [66]. Like our findings, mothers were more likely to develop mental health diagnosis. To our knowledge, this may be the only study that has shown a connection between pediatric scoliosis and siblings diagnosed with mental health disorders. It is important to acknowledge the limitation that some mental disorders like schizophrenia have a much stronger genetic component, which our data cannot further elucidate.

### Timing of mental health diagnosis

The article finds that the mean age of diagnosis of AIS was 13 years old and that the mean age of health disorder was diagnosed years after most often in their early to mid-20 s. AIS requires multidisciplinary management and a great deal of research is focused on perioperative and postoperative care [67]. This study finds higher rates of depression, body-image disturbances, and anxiety among these patients than the general US population. The delay in the development of multiple health disorders in early adulthood underscores the need to for primary care providers who see AIS patients to remain vigilant of the increased risk of developing mental health disorders years after their diagnosis of scoliosis, regardless of whether they received treatment.

### Limitation and strength

The nature of a survey study allowed for attaining responses across all 50 states. While it provides a large cohort, the survey design does not allow for specific queries into detailed review of patient’s charts. The study’s cross-sectional design does not demonstrate preoperative vs postoperative comparison of factors like quality of life or other psychiatric comorbidities. On the other hand, the fact most respondents were female may represent a bias as the prevalence does not reflect that of literature. Females do indeed represent the majority of AIS cases (ratio of 1.4:1 at lower Cobb angles to 7.2:1 at Cobb angle > 40 and are more likely to require serious management [3, 68, 69]. But we find that our ratio of respondents to be 9:1 female to male. Although ResearchMatch is connected to the NIH and used extensively across the country by private and academic institutions, institutions that do not use ResearchMatch may not be adequately captured thus demonstrating a referral bias. This limitation is compounded by the fact that treatment institution is unknown. Similarly, there may be a response bias in those who complete the survey. The population studied in this paper were majority AIS but included other causes which may introduce heterogeneity of the underlying etiology. The treatments such as use of preoperative traction and age at which treatment is initiated differ, which can affect these results. In addition, we are not provided with the operative details of these patients. Given the patient reported nature of this study, we cannot comment of further surgical factors that may affect outcomes. The degree of severity was not able to be quantitively measured, only categorically, due to heterogenous respondent reporting types. Finally, while the psychiatric comorbidities among pediatric scoliosis patients is higher, we acknowledge that these are multifactorial diseases that may be affected by factors not measured such as family history, education level, among other factors and cannot suggest causal relationship but only associations.

## Conclusion

In conclusion, we find that over half of pediatric patients with scoliosis have a psychiatric comorbidity, which may be diagnosed many years after the scoliosis diagnosis. The most prevalent psychiatric condition is depression, body-image disturbances, and anxiety. Given not only the physical but also psychosocial impact of scoliosis on these children, the authors recommend psychiatric screening for this population. In addition to illuminating new directions for research in the pediatric scoliosis, our findings generated concrete advice to pediatric teams and parents caring for these patients. Surgeons should consider incorporating holistic consideration for these patients and consider involving child psychiatric and mental health services preoperative and post-operatively. These findings highlight the importance of awareness of the psychiatric impact of scoliosis among pediatric patients, and potentially suggest a longer follow-up period to screen and treat comorbid mental health conditions.

## Data Availability

Data available on request from the authors.
